# Cardioprotection by Hypothyroidism Is Not Mediated by Favorable Hemodynamics—Role of Canonical Thyroid Hormone Receptor Alpha Signaling

**DOI:** 10.3390/ijms232113340

**Published:** 2022-11-01

**Authors:** Janina Pape, Helena Kerp, Helmut R. Lieder, Daniela Geist, Georg Sebastian Hönes, Lars C. Moeller, Petra Kleinbongard, Dagmar Führer

**Affiliations:** 1 Department of Endocrinology, Diabetes and Metabolism, University of Duisburg-Essen, 45122 Essen, Germany; 2 West German Heart and Vascular Center Essen, Institute for Pathophysiology, University of Essen Medical School, 45122 Essen, Germany

**Keywords:** cardioprotection, thyroid hormone receptor, ischemia/reperfusion injury, isolated heart, thyroid hormone, hypothyroidism

## Abstract

Hypothyroidism has been shown to reduce infarct size in rats, but the underlying mechanisms are unclear. We used isolated pressure-constant perfused hearts of control, hypothyroid and hyperthyroid mice and measured infarct size, functional parameters and phosphorylation of key molecules in cardioprotective signaling with matched heart rate. Compared with controls, hypothyroidism was cardioprotective, while hyperthyroidism was detrimental with enlarged infarct size. Next, we asked how thyroid hormone receptor α (TRα) affects ischemia/reperfusion (IR) injury. Thus, canonical and noncanonical TRα signaling was investigated in the hearts of (i) mice lacking TRα (TRα^0^), (ii) with a mutation in TRα DNA-binding domain (TRα^GS^) and (iii) in hyperthyroid TRα^0^ (TRα^0^hyper) and TRα^GS^ mice (TRα^GS^hyper). TRα^0^ mouse hearts were protected against IR injury. Furthermore, infarct size was reduced in the hearts of TRα^GS^ mice that lack canonical TRα signaling but maintain noncanonical TRα action. Hyperthyroidism did not increase infarct size in TRα^0^ and TRα^GS^ mouse hearts. These cardioprotective effects were not associated with increased phosphorylation of key proteins of *RISK*, *SAFE* and *eNOS* pathways. In summary, chronic hypothyroidism and the lack of canonical TRα signaling are cardioprotective in IR injury and protection is not due to favorable changes in hemodynamics.

## 1. Introduction

Thyroid hormones (THs) are crucial for normal cardiac function and orchestrate multifold effects in a healthy heart [1]. In humans, hypothyroidism is associated with an unfavorable cardiovascular risk profile, including dyslipidemia, bradycardia, endothelial dysfunction and hypertension, but no increased incidence of myocardial infarction (MI) [2]. In rodents, reduced infarct size has been observed in rats with chronic hypothyroidism [3], but the underlying mechanism(s) are still unclear. For example, the importance of the typically reduced heart rate and reduced left ventricular developed pressure (LVDP) in hypothyroidism and the potential role of classical protective signaling [4] for reduced infarct size have not been addressed. Furthermore, experimental studies on the outcome of myocardial ischemia/reperfusion (IR) injury in hypo- or hyperthyroid rats, focused on the evaluation of post-ischemic left ventricular function. The results were ambiguous as to whether THs are protective or detrimental in myocardial IR injury [3,5,6]. Infarct size is still the most robust endpoint of cardioprotection in preclinical studies [7,8] since the recovery of left ventricular function also depends on reversible injury (stunning) and the function of remote myocardium [9]. Although it is known that thyroid dysfunction affects transcriptional homeostasis and functional parameters of the heart, such as contractility or cardiac output [10] the precise role of THs in the diseased heart still requires clarification [1,2]. Specifically, in patients, the role of TH status in the outcome of MI has not yet been sufficiently investigated in randomized and controlled trials [1]. 

Cardiac TH effects are predominantly mediated via thyroid hormone receptor (TR) α [11,12]. However, it is not well understood how TR signaling precisely affects myocardial IR injury. While the involvement of TRα has been suggested, since in vivo inhibition of TRα after MI impaired post-ischemic left ventricular performance in mice [13,14,15,16], it remains unclear, which type of TRα signaling (canonical or noncanonical) is relevant for these processes. 

TRs belong to the superfamily of nuclear receptors, thus acting canonically as ligand-dependent transcription factors [17]. We and others have previously shown that TRs can also act independently of TH-target gene expression via rapid activation of cellular signaling pathways and voltage-activated potassium channels (noncanonical pathway) [18,19,20]. The mechanisms have been discussed in different reviews and also resulted in a new nomenclature for TH/TR action [17,21,22]. To distinguish between these two types of TR signaling, we generated a mouse model with a mutation in the TRα DNA-binding domain (TRα^GS^), which completely abrogates canonical TRα signaling while noncanonical signaling is preserved [19]. Consequently, the comparison of wildtype (WT), TRα knockout mice (TRα^0^) and TRα^GS^ mice allows differentiation between canonical and noncanonical TRα effects. 

**Figure 1:**
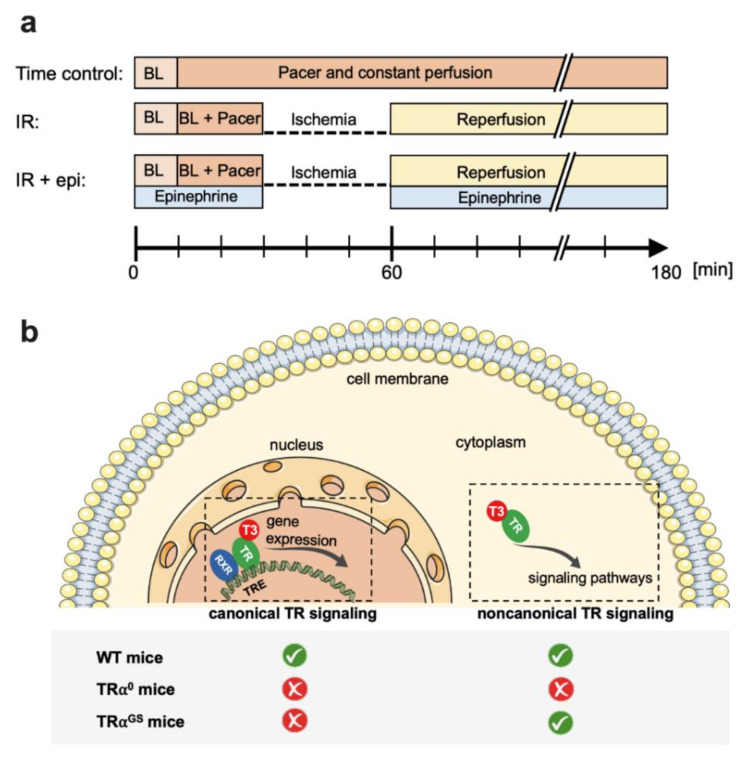
Overview of the experimental approach: (**a**) Timeline (minutes) of different perfusion protocols for investigation of isolated mouse hearts with the order of protocol components shown in boxes (BL = baseline perfusion without electrical atrial stimulation; BL + Pacer [500 bpm], epinephrine [23 µg/L]); Time control = constant perfusion; IR = 30 min global ischemia followed by 120 min reperfusion, IR + epi = 30 min global ischemia followed by 120 min reperfusion + epinephrine. (**b**) Overview of investigated mouse models with altered thyroid hormone receptor alpha (TRα) signaling: T3 = triiodothyronine; TR = thyroid hormone receptor; RXR = retinoid-x-receptor; TRE = thyroid responsive element; WT = wildtype; TRα^0^ = TRα knockout; TRα^GS^ = mutation in the TRα DNA-binding domain, which abrogates canonical but maintains noncanonical TRα signaling.

In the present study, we investigated the role of TH status in myocardial IR injury. Therefore, hearts of control, hypothyroid (hypo) and hyperthyroid (hyper) mice were isolated, pressure constant perfused and subjected to IR injury with matched heart rate by electrical atrial stimulation and matched LVDP in hypothyroid hearts by epinephrine administration (Figure 1a). In a second approach, the mode of TRα signaling in myocardial IR injury was investigated by using TRα^0^, TRα^0^hyper, TRα^GS^, TRα^GS^hyper and respective WT mouse hearts (Figure 1b). Infarct size and left ventricular function as well as phosphorylation of key proteins in classical cardioprotective signaling pathways were investigated as previously described [4,7,8]. 

## 2. Results

### 2.1. Serum TH Status and Heart Rate in Mice with Thyroid Dysfunction

**Figure 2:**
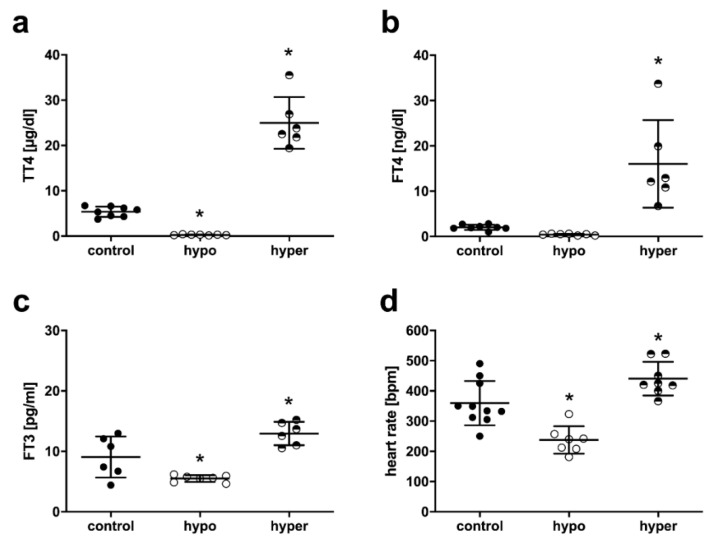
Thyroid hormone serum concentrations and ex vivo heart rate of mice with manipulation of thyroid function. Serum concentration of (**a**) total thyroxine (TT4), (**b**) free thyroxine (FT4), and (**c**) free thyronine (FT3) in euthyroid (control), hypothyroid (hypo) and hyperthyroid (hyper) mice, respectively; *n* = 6–8; Data are means ± standard deviations; * *p* < 0.05 vs. control. (**d**) Ex vivo heart rate in [beats per minute (bpm)] in isolated pressure constant perfused hearts of euthyroid (control), hypothyroid (hypo), hyperthyroid (hyper) mice, respectively; *n* = 7–10; Data are means ± standard deviations; * *p* < 0.05 vs. control.

To confirm the successful induction of thyroid dysfunction in male C57BL/6JRj mice, TH serum concentrations were measured. Treatment of mice with anti-thyroidal drugs (0.02% methimazole (MMI), 0.5% perchlorate (ClO_4_^−^) and a low iodine diet (LoI)) decreased TT4 and FT3 in hypothyroid (hypo) compared with control mice. In contrast, TH treatment with 1µg/mL T4 increased concentrations of serum TT4, FT4 and FT3 in hyperthyroid (hyper) compared with control mice (Figure 2a–c). The baseline heart rate was reduced in the hearts of hypo and elevated in the hearts of hyper mice compared with controls (Figure 2d).

### 2.2. Impact of Thyroid Dysfunction on Infarct Size

**Figure 3:**
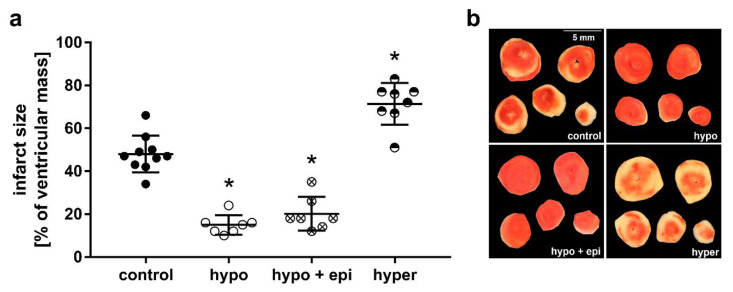
Myocardial infarct size of isolated pressure constant perfused euthyroid mouse hearts (control), hypothyroid mouse hearts in absence (hypo) and in presence of epinephrine (hypo + epi [23 µg/L]), and hyperthyroid (hyper) mouse hearts, respectively. (**a**) Infarct size in [% of ventricular mass] and (**b**) representative heart slices after 120 min of reperfusion and triphenyltetrazoliumchloride (TTC) staining, respectively; *n* = 7–10; Data are means ± standard deviations; * *p* < 0.05 vs. control.

Thirty min of global ischemia and 120 min of reperfusion resulted in an infarct size of 48 ± 9% of the ventricular mass in the hearts of control mice (Figure 3). Hypothyroidism in absence of epinephrine reduced infarct size to 15 ± 5% and in presence of epinephrine to 20 ± 8% of the ventricular mass (*p* < 0.05), while hyperthyroidism enlarged infarct size to 71 ± 10% of the ventricular mass (*p* < 0.05), respectively (Figure 3). In time controls (TC) that did not undergo IR, only negligible infarction was detected (Figure S1). In summary, chronic thyroid hormone deprivation in hearts led to smaller infarcts, while thyroid hormone excess led to larger infarcts compared with euthyroid controls. 

### 2.3. Impact of Thyroid Dysfunction on Hemodynamics

To evaluate the functional parameters of the hearts throughout IR, we recorded the coronary flow (CF) and left ventricular developed pressure (LVDP) at different time points. At baseline, where isolated mouse hearts were initially perfused without electrical atrial stimulation, CF and LVDP were decreased in hypo, while LVDP was increased in hyper compared with control mouse hearts (Table 1). At the pacer baseline, CF and LVDP were also decreased in hypo, while CF and LVDP were comparable between hyper and control mice. 

To correct for a pacing-induced reduction in LVDP at baseline in hypothyroid hearts (Table 1), which could have induced cardioprotection per se, epinephrine was continuously added to the perfusion buffer to increase LVDP in hypothyroid hearts (hypo + epi). Of note, hypo mouse hearts in the absence and presence of epinephrine showed preserved left ventricular function after IR injury, as there was no difference in LVDP at reperfusion in comparison to LVDP at pacer baseline (Table 1). In contrast, compared with the pacer baseline, LVDP recovered less at reperfusion in control and hyper mouse hearts, while CF during reperfusion was comparable in the two groups (Table 1). CF and LVDP were stable over time in time controls (Table S1).

### 2.4. Impact of Thyroid Dysfunction on Cardioprotective Signaling Pathways

To address a possible involvement of signal transduction pathways previously described in the context of cardioprotection [4], i.e., reperfusion injury salvage kinase- (*RISK)*, the survivor activating factor enhancement- (*SAFE*) and the nitric oxide/protein kinase G pathway (the latter referred to as *eNOS* pathway), expression and phosphorylation of the following key proteins were investigated in mouse hearts after ischemia–reperfusion: AKT_Ser473_, ERK1/2_Thr202/Tyr204,_ STAT3_Tyr705_, p38 MAPK_Thr180/Tyr182_ and eNOS_Ser1177_.

**Figure 4:**
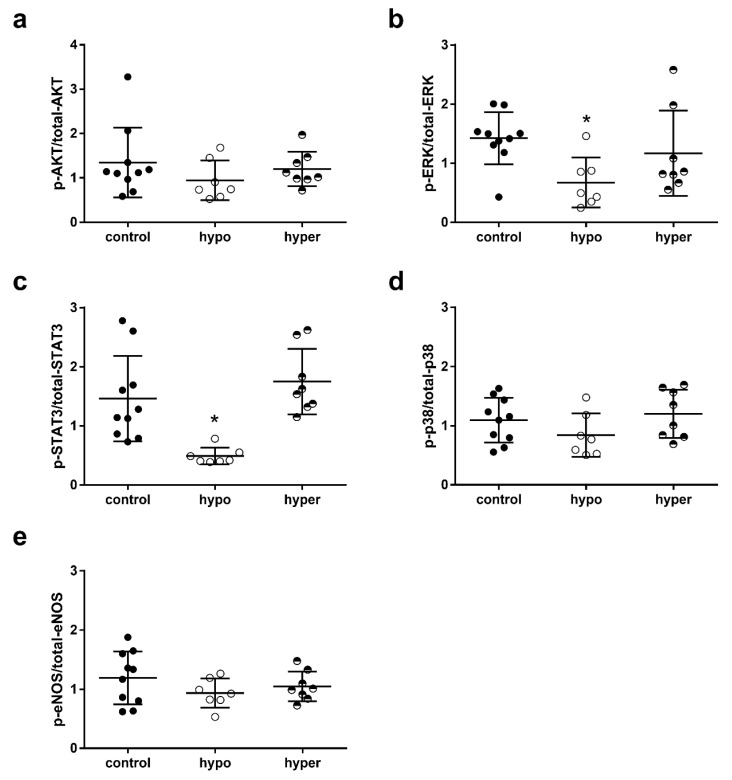
Phosphorylation of key proteins of classical cardioprotective proteins in euthyroid (control), hypothyroid (hypo) and hyperthyroid (hyper) mouse hearts after 30 min of ischemia and 120 min of reperfusion: (**a**) protein kinase B (p-AKT/total-AKT), (**b**) extracellular-signal-regulated kinases (p-ERK/total-ERK), (**c**) signal transducer and activator of transcription 3 (p-STAT3/total-STAT3), (**d**) p38-mitogen-activated protein kinase (p-p38/total-p38) and (**e**) endothelial nitric oxide synthase (p-eNOS/total-eNOS). The phosphorylation of proteins was normalized to the respective total protein; *n* = 7–10; Data are means ± standard deviations; * *p* < 0.05 vs. control.

Change in TH status itself influenced total protein expression (Figure S2). Compared with euthyroid controls total-AKT, ERK1/2, STAT3, p38 MAPK and eNOS were increased in hypothyroid hearts, total ERK1/2 and eNOS were increased in hyperthyroid hearts and total-p38 MAPK was decreased. Phosphorylation levels of AKT_Ser473_ (p-AKT/total-AKT), p38 MAPK (p-p38/total-p38) and eNOS_Ser1177_ (p-eNOS/total-eNOS) were comparable in hearts of all three mouse groups (Figure 4a,d,e). Phosphorylation of ERK1/2_Thr202/Tyr204_ (p-ERK/total-ERK) and STAT3_Tyr705_ (p-STAT3/total-STAT3) were significantly reduced (both *p* < 0.05) in hypo mouse hearts and TH treatment restored phosphorylation compared with controls (Figure 4b,c). In summary, chronic thyroid dysfunction with subsequent changes in mouse infarct size after IR injury was not accompanied by increased phosphorylation of key proteins of classical cardioprotective pathways. Under hypothyroidism, infarcts were smaller and phosphorylation of ERK, and STAT3 in mouse hearts was decreased. Larger infarcts were found under hyperthyroidism associated with restored phosphorylation of ERK and STAT3. 

### 2.5. Serum TH Status and Heart Rate in Mice with Altered TRα Signaling 

**Figure 5:**
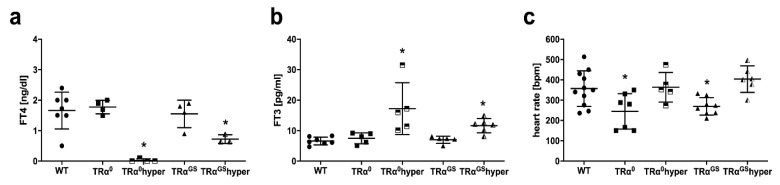
Thyroid hormone serum concentrations and ex vivo heart rate of transgenic mice. Serum concentrations of (**a**) free thyroxine (FT4) and (**b**) free thyronine (FT3) of wildtype (WT), TRα^0^, hyperthyroid TRα^0^ (TRα^0^hyper), TRα^GS^ and hyperthyroid TRα^GS^ (TRα^GS^hyper) mice, respectively; *n* = 4–7; Data are means ± standard deviations; * *p* < 0.05 vs. WT. (**c**) Ex vivo heart rate in [beats per minute (bpm)] in isolated pressure-constant perfused hearts of wildtype (WT), TRα^0^, hyperthyroid TRα^0^ (TRα^0^hyper), TRα^GS^ and hyperthyroid TRα^GS^ (TRα^GS^hyper) mice, respectively; *n* = 5–11; Data are means ± standard deviations; * *p* < 0.05 vs. WT.

To clarify the role of TRα signaling during IR injury, isolated hearts of WT mice, mice lacking TRα (TRα^0^) and mice expressing a mutant TRα (TRα^GS^) that is incapable of DNA binding were used. TRα^0^ and TRα^GS^ mice were kept on the same genetic background (C57BL/6J) to assure comparability between the different strains. In addition, to distinguish between indirect TH effects on the heart and direct TRα dependent effects, hyperthyroidism was induced by treatment with 400 ng/mL T3 in an additional cohort of TRα (TRα^0^hyper) and TRα^GS^ mice (TRα^GS^hyper). FT4 and FT3 serum concentrations were comparable between TRα^0^, TRα^GS^ and WT mice (Figure 5a,b). As expected, T3 treatment resulted in increased serum FT3 concentrations in TRα^0^hyper and TRα^GS^hyper mice compared with WT mice (Figure 5b), while FT4 concentrations were decreased (Figure 5a). Baseline heart rate was reduced in TRα^0^ and TRα^GS^ mouse hearts compared with WT, while heart rate was comparable between WT, TRα^0^hyper and TRα^GS^hyper mouse hearts, respectively (Figure 5c).

### 2.6. Impact of TRα Signaling on Infarct Size 

**Figure 6:**
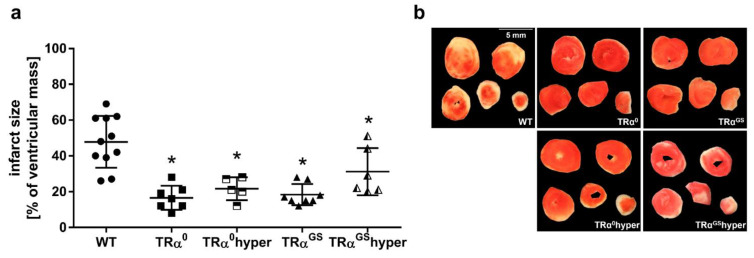
Myocardial infarct size of isolated pressure constant perfused hearts of wildtype (WT), TRα^0^, hyperthyroid TRα^0^ (TRα^0^hyper), TRα^GS^ and hyperthyroid TRα^GS^ (TRα^GS^hyper) mice, respectively. (**a**) Infarct size in [% of ventricular mass] and (**b**) representative heart slices after 120 min of reperfusion and triphenyltetrazoliumchloride (TTC) staining, respectively; *n* = 5–11; Data are means ± standard deviations; * *p* < 0.05 vs. WT.

The lack of TRα in TRα^0^ mouse hearts resulted in a reduced infarct size of 17 ± 7% of ventricular mass compared with WT (51 ± 14%) (*p* < 0.05). In contrast to the hyper group (Figure 3), systemic hyperthyroidism in TRα^0^ mice did not result in enlarged infarct size in TRα^0^ mouse hearts (TRα^0^ 17 ± 7 vs. TRα^0^hyper 22 ± 6% of the ventricular mass, n.s.). Importantly, the hearts of TRα^GS^ and hyperthyroid TRα^GS^ mice were also protected against IR injury with an infarct size of 18 ± 6% and 31 ± 13% of the ventricular mass, respectively, compared with WT (*p* < 0.05) (Figure 6). Taken together, hypothyroidism and the lack of canonical TRα signaling, absent in TRα^0^ and TRα^GS^ mice, resulted in reduced infarct size. 

### 2.7. Impact of TRα Signaling on Hemodynamics

At baseline CF and LVDP were comparable between WT, TRα^0^, TRα^0^hyper, TRα^GS^ and TRα^GS^hyper mouse hearts (Table 1). At pacer baseline, LVDP in TRα^0^ mouse hearts was lower compared with WT hearts, while it was comparable between TRα^GS^, TRα^0^hyper, TRα^GS^hyper and WT (Table 1). During reperfusion, the recovery of LVDP was improved in TRα^0^, TRα^0^hyper, TRα^GS^ and TRα^GS^hyper compared with WT, reaching significance in TRα^0^, TRα^0^hyper and TRα^GS^ mouse hearts. Compared with the pacer baseline, LVDP at reperfusion decreased in WT and in TRα^0^hyper and TRα^GS^hyper mouse hearts, while LVDP at reperfusion was comparable to LVDP at pacer baseline in TRα^0^ and TRα^GS^ mouse hearts. CF during reperfusion was comparable in all groups (Table 1). 

### 2.8. Impact of Mode of TRα Signaling on Phosphorylation of Key Proteins of RISK, SAFE and eNOS Pathway in Mouse Hearts after Ischemia–Reperfusion

**Figure 7:**
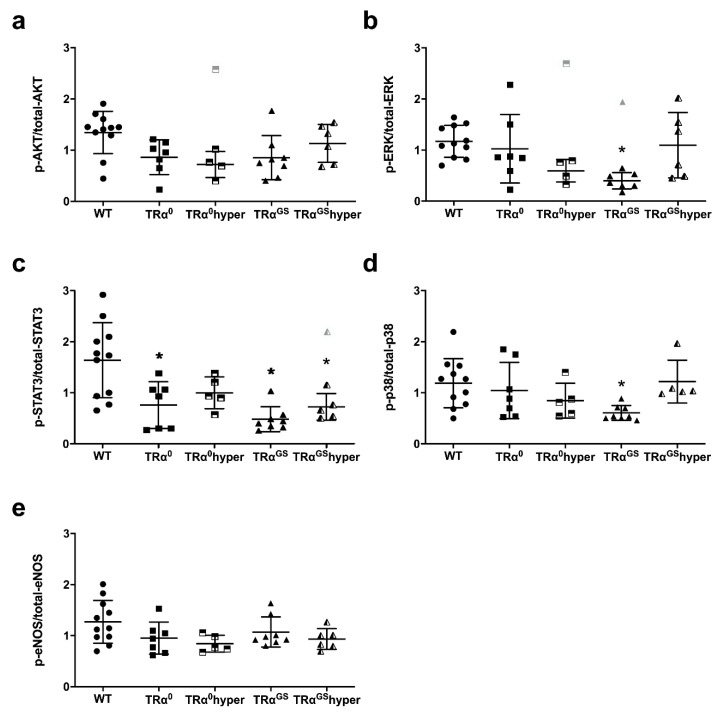
Phosphorylation of key proteins of classical cardioprotective proteins in wildtype (WT), TRα^0^, hyperthyroid TRα^0^ (TRα^0^hyper), TRα^GS^ and hyperthyroid TRα^GS^ (TRα^GS^hyper) mice. (**a**) protein kinase B (p-AKT/total-AKT), (**b**) extracellular-signal-regulated kinases (p-ERK/total-ERK), (**c**) signal transducer and activator of transcription 3 (p-STAT3/total-STAT3), (**d**) p38-mitogen-activated protein kinase (p-p38/total-p38) and (**e**) endothelial nitric oxide synthase (p-eNOS/total-eNOS) after 30 min of ischemia and 120 min of reperfusion. The phosphorylation of proteins was normalized to the respective total protein; *n* = 5–11; Data are means ± standard deviations; * *p* < 0.05 vs. WT; significant outliers are shown in light gray.

Phosphorylation of AKT_Ser473_ (p-AKT/total-AKT) and eNOS_Ser1177_ (p-eNOS/total-eNOS) in mouse heart did not differ between genotypes and treatment regimens (Figure 7a,e). Reduced phosphorylation of ERK1/2_Thr202/Tyr204_ (p-ERK/total-ERK) and STAT3_Tyr705_ was detected in the hearts of mice with altered TRα signaling compared with WT, with a significant decrease for ERK1/2_Thr202/Tyr204_ in TRα^GS^ and for STAT3_Tyr70_ in TRα^0^, TRα^GS^ and TRα^GS^hyper groups (all *p* < 0.05; Figure 7b,c). Moreover, reduced phosphorylation was observed for p38 MAPK in the hearts of TRα^GS^ mice (*p* < 0.05, Figure 7d). Data on total protein expression in the hearts of the different genotypes and treatment regimens are shown in Figure S3. 

In summary, altered TRα signaling due to loss of the receptor in TRα^0^ or loss of canonical signaling in TRα^GS^ mice was consistently associated with reduced infarct size, but not increased phosphorylation of key proteins of classical cardioprotective pathways. Notably, similar changes in phosphorylation of key signaling proteins of *RISK* and *SAFE* pathways were found in mice with altered TRα signaling as in mice with induced hypothyroidism. 

## 3. Discussion

To clarify the impact and mode of TH/TR signaling during myocardial IR injury, we investigated infarct size, left ventricular function and classical cardioprotective signaling pathways in isolated perfused hearts of mice with induced changes in TH status and in transgenic mice with altered TRα signaling (Figure 1). We found that chronic hypothyroidism confers cardioprotection and show that this is not a result of more favorable hemodynamics under TH deficiency. Furthermore, we demonstrate for the first time the pivotal role of canonical TRα signaling for cardioprotection while noncanonical TRα signaling preserves left ventricular pressure. Interestingly the observed protection from IR injury in the hearts of hypothyroid mice and transgenic mice with altered TRα signaling was not paralleled by an increase in phosphorylation of key proteins of *RISK*, *SAFE* and *eNOS* pathways at the end of 120 min reperfusion [4,23]. Hence, through which cellular and subcellular signaling pathway(s) the lack of canonical TRα action confers cardioprotection is open to future studies. 

### 3.1. Hypothyroidism and Lack of Canonical TRα Signaling Confer Cardioprotection in the Absence of Favorable Hemodynamics and without Canonical Protective Signaling

Recently, hypothyroidism has been shown to reduce infarct size in rats [3]. However, in that study, heart rate was not matched between groups. Nevertheless, this is an important prerequisite to generate comparable experimental conditions [7], since reduced heart rate and LVDP may influence IR injury outcome per se. Thus, we matched heart rate by electrical atrial stimulation and LVDP by adding epinephrine to hypothyroid hearts. Our data now show that cardioprotection by hypothyroidism is independent of favorable hemodynamics during IR, as infarct size was reduced and LVDP was preserved in hypothyroid hearts in the absence and also in presence of epinephrine, which is in line with previous reports in rat heart [3,24]. A recent study suggested that the higher tolerance to IR injury under hypothyroidism is linked to an increased stoichiometric ratio of two mitochondrial uniplex subunits and the threshold to cytosolic Ca^2+^, suggesting a reduced Ca^2+^ content in mitochondria which could result in more tolerance to Ca^2+^ overload and thus delaying mPTP opening and mitochondrial dysfunction [25]. In our study, preceding hyperthyroidism increased infarct size and decreased functional recovery of LVDP compared with baseline. This is consistent with studies in rats, where left ventricular functional recovery was impaired in hyperthyroid hearts [6]. Others reported a cardioprotective phenotype under hyperthyroidism with improved recovery of LVDP [26,27], but infarct size was not determined. Of note, infarct size is still the most robust endpoint of cardioprotection in preclinical studies [7,8], since the recovery of left ventricular function also depends on reversible injury (stunning) and the function of remote myocardium [9,28]. Hence, our assessment of infarct sizes underscores that chronic hypothyroidism is protective while hyperthyroidism is not in IR injury. The latter is consistent with the increased cardiovascular morbidity and mortality in hyperthyroid patients documented in epidemiological studies [1,29]. Moreover, our results—for the first time—demonstrate a heart rate- and LVDP-independent cardioprotective effect of hypothyroidism. Although hypothyroidism is generally associated with unfavorable changes in a number of cardiovascular risk factors in humans, our data show in the acute situation hypothyroidism is more favorable than hyperthyroidism to a degree that preceding hypothyroidism can be considered protective. Thus, hypothyroidism and its influence on general cardiovascular risk factors ultimately leading to ischemia and impact of thyroidal state when ischemia occurs need to be distinguished. 

Others have shown that treatment of rats with a selective TRα inhibitor impaired the functional performance of isolated rat hearts after ischemia [13]. However, it remained unclear by which signaling mode, canonical or noncanonical, TRα affects myocardial infarct size. Here, we demonstrate, that global TRα deficiency in TRα^0^ mouse hearts is cardioprotective, and furthermore, that lack of TRα also protects against the detrimental effect of systemic hyperthyroidism on IR injury. Strikingly, the cardioprotective effect was still present in isolated mouse hearts of TRα^GS^ and even of hyperthyroid TRα^GS^ mice, a mouse model with abrogated DNA-binding of TRα. After IR, infarct size was reduced and LVDP recovery was improved, emphasizing cardioprotection under hypothyroidism. Thus, we suggest that lack of canonical TRα signaling confers cardioprotection and that noncanonical TRα action, while relevant to other aspects of cardiovascular function [19], is not involved in this process. 

An unexpected finding was that the observed protection from IR injury was not reflected in an increase in phosphorylation in key proteins of described classical cardioprotective pathways in the hearts of our mouse models. For example, increased phosphorylation of STAT3 has been shown in ischemic conditioning-induced cardioprotection in the non-TH context [30,31]. We found decreased phosphorylation of STAT3, in the hearts of hypothyroid mice and in transgenic mice with altered TRα signaling. In our view, it is unlikely that this decrease in STAT3 phosphorylation is causally involved in infarct size reduction by hypothyroidism or the lack of canonical TRα signaling. Rather, we suggest that the decrease in STAT3 phosphorylation could be associated with chronic TH deprivation since physiological levels of TH have been shown to promote the phosphorylation of STAT3 in vitro [32]. Hence, TH could contribute to the maintenance of myocardial STAT3 signaling via TRα in IR injury. We also found no correlation of phosphorylation of p38 MAPK or eNOS with reduced infarct sizes in our study, suggesting that these pathways are not responsible for the observed cardioprotective TH effects. 

One limitation of our study is that protein expression and phosphorylation were only analyzed at the end of 120 min of reperfusion, while activation at early time points was not assessed. Thus, we may have missed an important time window. In addition, inhibitor experiments to conclusively demonstrate or refute the involvement of single cardioprotective pathways were not conducted. However, our data showing an association between reduced infarct sizes and the consistent absence of activation of RISK, SAFE, and eNOS pathways in the hearts of hypothyroid mice and mice with the abrogation of canonical TRα signaling could also suggest that TH-related cardioprotection is different from classical cardioprotection [30,31]. Clearly, future studies are warranted to address these issues, in particular, the timing and blockade of pathway activation and the role of subcellular, e.g., mitochondrial TRα signaling with the aim to ultimately understand how TH-dependent cardioprotection is achieved on the molecular level. 

### 3.2. Noncanonical TRα Signaling Contributes to Baseline left Ventricular Pressure

Our study shows that LVDP and CF were decreased in isolated hypothyroid hearts and that LVDP was decreased in TRα^0^ hearts at pacer baseline. Accordingly, an altered cardiac structure as well as a decrease in cardiac contractility could be found in mice with a deletion of TRα [33]. Moreover, contractile function and transcript levels of genes encoding contractile proteins, e.g., myosin heavy chain (MHC) α were decreased in TRα knockout mice [19,34]. In agreement with this, cardiac dysfunction with a reduction in left ventricular pressure was previously found in MHC mutant mouse hearts [35]. Strikingly, and in contrast to TRα^0^, hearts of TRα^GS^ mice showed a normal LVDP similar to WT hearts, demonstrating that presence of noncanonical TRα signaling is sufficient to preserve LVDP. Hence, noncanonical TRα signaling contributes to a normal LVDP under euthyroidism, while the lack of noncanonical TH/TRα action contributes to decreased LVDP in hypothyroid and TRα^0^ mouse hearts. 

We also noted that hyperthyroidism increased LVDP at baseline. This is consistent with an increase in cardiac contractility after TH treatment in rats [3]. Interestingly and contrary to our expectations, the hearts of TRα^0^hyper mice exhibited a normal LVDP at the level of WT mice, suggesting a possible involvement of TRβ signaling under hyperthyroidism on cardiac contractility. Thus, regulation of cardiac contractility and functional recovery after IR injury involve different modes of TH signaling. 

### 3.3. Lack of Canonical TRα Signaling Determines Bradycardia

Both systemic hypothyroidism and the lack of canonical TRα signaling decreased ex vivo heart rate in our study. This is in line with a decreased heart rate as classical clinical findings in patients with hypothyroidism [1,2] and with TRα being the predominant TR isoform for TH action in murine hearts [36]. Consistently, TRα has been shown to regulate the expression of action potential repolarization and pacemaker channels HCN2 and HCN4 [34]. In addition, while our in vivo data indicated that noncanonical TRα signaling contributes to extrinsic regulation of heart rate, ex vivo heart rate was decreased in TRα^GS^ and TRα^0^ compared with WT [19]. Considering that TRα^0^ and TRα^GS^ mice display a hypothyroid gene expression pattern in the heart [19], we suggest that bradycardia is the result of a hypothyroid-like myocardium due to the lack of canonical TRα signaling and thus an intrinsic effect.

Previously, it was reported that bradycardia in TRα1-deficient mice could be partly reversed by T3 treatment yet did not equal the heart rate of controls [37], suggesting either an influence of the autonomous nervous system or compensation through TRβ for the lack of TRα in the heart. In our ex vivo model, we found that heart rates of hyperthyroid TRα^0^ and TRα^GS^ mouse hearts were comparable to WT mouse hearts, indicating that heart intrinsic residual TRβ effects may partially compensate for the absence of TRα. Ultimately, studies of TRβ knockout and TRβ^GS^ mouse hearts would be necessary to confirm the possible influence of TRβ on different cardiac readout parameters. In particular, organ- and ultimately cell-specific transgenic variants would be the mouse models of choice to exclude systemic side effects of respective global knockout/knockin mice and to truly reflect local TH action in the heart.

## 4. Materials and Methods

### 4.1. Mice and Treatment

All animal experiments were performed in accordance with the German regulations for Laboratory Animal Science (GVSOLAS) and the European Health Law of the Federation of Laboratory Animal Science Associations (FELASA). The protocols for animal studies were approved by the *Landesamt für Natur, Umwelt und Verbraucherschutz Nordrhein-Westfalen* (LANUV-NRW), Germany (AZ: 84-02.2014.A092, 84-02-2017.A157 and 84-02.04.2016.A261). Male C57BL/6JRj mice (Janvier Labs, Laval (Mayenne), France) aged 3–6 months (*n* = 5–10 animals/treatment) were used for induction of thyroid dysfunction. Animals were housed in temperature- (23 ± 1 °C) and light-controlled (inverse 12:12 h light-dark cycle) conditions. Food and water were provided ad libitum. For experiments on thyroid dysfunction, chronic hyperthyroidism was induced by adding 1 μg/mL T4 to the drinking water (T4 was dissolved in 40 mmol/L NaOH and 0.1% bovine serum albumin (BSA)). For induction of chronic hypothyroidism, animals were fed a low-iodine diet (LoI) and received drinking water supplemented with 0.02% methimazole (MMI), 0.5% perchlorate (ClO_4_^−^) and 0.3% saccharine as a sweetener. Control and hyperthyroid animals were fed a control diet (LoI with added potassium iodide). The treatment period was 3 weeks [38,39]. 

Generation of TRα^0^ and TRα^GS^ mice, based on background strain C57BL/6J mice, was previously described [19]. Wildtype littermates were used as respective controls. Chronic hyperthyroidism in TRα^0^ and TRα^GS^ mice was induced by adding 400 ng/mL 3,3′,5-Triiodothyronine (T3) (Sigma-Aldrich, St. Louis, MO, USA) to the drinking water (T3 was dissolved in 40 mmol/L NaOH and 0.1% bovine serum albumin) (Sigma-Aldrich (A7906), St. Louis, MO, USA). The treatment period was 3 weeks.

### 4.2. Isolated Mouse Hearts

The experimental protocols, measurements of coronary flow (CF), left ventricular pressure (LVP) and quantification of infarct size were standardized [7,8] and described in detail before [40,41]. After cervical dislocation, 200 IU of heparin (Heparin-Natrium-2500-Ratiopharm, Ratiopharm GmbH, Ulm, Germany) were injected intraperitoneally to prevent coagulation, and hearts were rapidly excised. Within two minutes, hearts were cannulated (mouse heart cannula, Hugo Sachs Elektronik, March, Germany) under a stereomicroscope (LS 6000IC, Beckman Coulter, Krefeld, Germany) through the aorta in ice-cold 0.9% NaCl. Hearts were perfused in Langendorff mode [7] with a modified Krebs–Henseleit buffer (Table S2). The perfusion pressure was 80 mmHg and continuously monitored above the aortic cannula using a transducer (CODAN pvb Medical, Lensahn, Germany). The perfusate temperature was held constant by a heat exchanger located next to the aortic cannula. Coronary flow (CF) was measured by an in-line ultrasonic flow probe (TS410, Transonic Systems Inc., Ithaca, NY, USA) connected to the aortic cannula. A fluid-filled cling film balloon was inserted through the mitral valve into the left ventricular cavity and connected to a pressure transducer to allow continuous monitoring of LVP. The left ventricular end-diastolic pressure was set to 5 to 15 mmHg at baseline. Spontaneous heart rate was determined within the first ten minutes. Hearts were then paced to 500 beats/min (DPT-6000, Pvb Codan, Forstinning, Germany) by right atrial electrical stimulation. CF and end-diastolic and peak LVP were continuously recorded (LabChart 8, LabChart, ADInstruments Pty Ltd., Sydney, New South Wales, Australia). Left ventricular developed pressure (LVDP) was calculated as the difference between maximal and minimal LVP [40]. LVDP and CF were calculated as mean values during the last minute of the baseline (10 min), at the end of the pacer baseline (30 min), at the beginning of ischemia (5 min), at the end of ischemia (25 min), and at 10, 20, 30, 40, 50 and 60 min of reperfusion. Time points were chosen to allow comparison with existing cardiology studies [3,5,6,7]. The temperature of the humidified organ chamber was continuously kept between 37.0 and 37.5 °C to avoid hypothermia during ischemia. 

### 4.3. Protocols for Isolated Mouse Hearts (Figure 1) 

*Global IR* [8]: After a stabilization period of 30 min (10 min baseline and 20 min pacer baseline), hearts were subjected to 30 min global no-flow ischemia followed by 120 min reperfusion. 

*Global IR + epinephrine*: One group of hypothyroid mouse hearts received 23 µg/L epinephrine (Suprarenin, Sanofi, Paris, France) via syringe pump during a stabilization period of 30 min and during 120 min of reperfusion to compare LVDP at reperfusion with LVDP at baseline for evaluation of functional recovery after ischemia. 

*Time controls*: Control hearts were perfused for a duration period equal to the experimental protocol, i.e., 180 min but without ischemia. 

### 4.4. Infarct Size Determination

After 120 min reperfusion, hearts were frozen at −20 °C overnight and cut into 4–6 transverse 1 mm thick slices. The slices were immersed in 2,3,5-triphenyltetrazolium chloride (TTC) solution 1% (*w*/*v*) dissolved in phosphate buffer, consisting of 77.4% (*v*/*v*) 0.1 mol/L Na_2_HPO_4_ and 22.6% (*v*/*v*) 0.1 mol/L NaH_2_PO_4_, and incubated in a water bath at 37 °C for 5 min. Stained slices were photographed from both sides and were quickly frozen in liquid nitrogen and stored at −80 °C for later analysis. Total slice area and areas of viable tissue (red) and necrotic tissue (white) were quantified by computerized planimetry (ImageJ 1.48 v, National Institutes of Health, Bethesda, Maryland). Infarct size was calculated as a percent of the sum of left and right ventricular mass (% of ventricular mass).

### 4.5. Serum TH Status

After heart excision, blood samples from the abdominal caval vein were harvested. Total thyroxine (TT4), free thyroxine (FT4) and free thyronine (FT3) serum concentrations were measured using commercial ELISA kits according to the manufacturer’s instructions (DRG Instruments GmbH, Marburg, Germany; minimum detectable TH concentrations: 0.5 µg/dl for TT4, 0.05 ng/dl for FT4 and 0.05 pg/mL for FT3). Serum samples with known TH concentrations were used as controls and TH serum concentrations were normalized to corresponding wildtypes.

### 4.6. Immunoblot Analysis

Snap-frozen ventricular samples from the middle heart slice were homogenized in 100 mmol/L tris(hydroxymethyl)aminomethane (TRIS) with 2% sodium dodecyl sulfate (SDS; w/V; SERVA Electrophoresis GmbH, Heidelberg, Germany), heated to 70 °C for 5 min and centrifuged at 14,000 g for 10 min. The protein lysate-containing supernatant was stored at −80 °C in aliquots to prevent freeze- and thaw cycles. Proteins were separated by electrophoresis on precasted SDS-polyacrylamide gels (BioRad, Munich, Germany) and transferred to polyvinylidene fluoride membranes (BioRad, Munich, Germany). Membranes were stained with Ponceau S (SERVA, Heidelberg, Germany) as loading/transfer control. After blocking with fat-free milk (BioRad, Munich, Germany), membranes were incubated with antibodies directed against the phosphorylated form of endothelial nitric oxide synthase (eNOS_ser1177_) (Santa Cruz #81510, mouse monoclonal), protein kinase B (AKT_ser473_) (Cell Signaling #9271, mouse polyclonal), extracellular-signal-regulated kinases (ERK_thr202/tyr204_) (Cell Signaling #9101, rabbit polyclonal), signal transducer and activator of transcription 3 (STAT3_tyr705_) (Cell Signaling #9138, mouse monoclonal) and p38-mitogen-activated protein kinase (p38 MAPK_thr180/tyr182_) (Cell Signaling #9211, rabbit polyclonal). After incubation with the respective secondary antibody, immunoreactive signals were detected by chemiluminescence (Pierce Biotechnology, Waltham, MA, USA) and quantified with ChemCam/LabImage1D software (INTAS, Göttingen, Germany). Membranes were stripped and re-probed for the detection of the respective total form of eNOS protein (BD Biosciences, #610296, mouse monoclonal), AKT protein (Cell Signaling #9272, rabbit polyclonal), ERK protein (Cell Signaling #9102, rabbit polyclonal), STAT3 protein (Cell Signaling #9139, mouse monoclonal) and p38 MAPK protein (Cell Signaling #9212, rabbit polyclonal). The immunoreactivity of phosphorylated proteins was normalized to the immunoreactivity of the respective total protein which, in turn, was normalized to Ponceau S staining (Figure S4 and S5).

### 4.7. Statistical Analysis 

Investigators analyzing infarct size and protein expression were blinded for genotype and treatment group. Data are presented as means ± standard deviations. Data were tested for normality with the Kolmogorov–Smirnov test (GraphPad Prism 6 (GraphPad, San Diego, CA, USA)). Data sets with a normal distribution (TH serum concentrations of mice with thyroid dysfunction, heart rate, infarct size, CF and LVDP at baseline, protein expression) were analyzed using one-way ANOVA with Tukey`s post hoc test (comparison of three or more groups). Two-way ANOVA for repeated measures with Bonferroni’s post hoc test was used for normally distributed data sets on CF and LVDP in isolated mouse hearts with 120 min reperfusion (time point and group). Data sets without normal distribution (TH serum concentrations of transgenic mice) were corrected for multiple comparisons with Kruskal–Wallis-Test (GraphPad Prism 6 (GraphPad, San Diego, CA, USA). Differences were considered significant at the level of *p* < 0.05.

## 5. Conclusions

Taken together, our results comprehensively show that lack of canonical TRα signaling confers cardioprotection, similarly to systemic hypothyroidism independent of favorable hemodynamics and that local TH action in the heart determines the cardioprotective phenotype, as TRα^0^hyper and TRα^GS^hyper mouse hearts were also protected against IR injury. In addition, we demonstrate that noncanonical TRα signaling contributes to normal LVDP. Furthermore, the lack of canonical TRα signaling is causal for inducing intrinsic bradycardia or, vice versa, canonical TRα signaling is necessary to maintain intrinsic heart rate. However, under hyperthyroid conditions, TRβ might also play a role in intrinsic heart rate regulation and cardiac contractility. 

In conclusion, both, canonical and noncanonical TR signaling pathways are required and determine different heart function aspects. Our results emphasize the crucial role of TH/TR signaling in IR injury and necessitate clarification of cell-specific requirements in this setting to make it an attractive avenue for advanced cardioprotective therapy.

## Figures and Tables

**Table 1:** Coronary flow and left ventricular developed pressure in isolated mouse hearts under different perfusion protocols for investigation of effects of hypo- and hyperthyroidism and mode of TRα signaling on ischemia–reperfusion injury. Coronary flow (CF) and left ventricular developed pressure (LVDP) of mouse hearts were analyzed at different time points: at baseline, at pacer baseline, at 5 and 25 min of ischemia (isch5, isch25) and at 10, 20, 30, 40, 50, 60 min of reperfusion (rep10-rep60); *n* = 5–11; Data are means ± standard deviations. * *p* < 0.05 vs. control and WT, respectively, # *p* < 0.05 vs. pacer baseline; § = pacer baseline + epinephrine [23 µg/L]; controls, hypo and hyper = hearts of euthyroid, hypothyroid and hyperthyroid mice; epi = epinephrine; WT, TRα^0^, TRα^GS^ = hearts of wildtype, TRα knockout and mice expressing a TRα^GS^ that is incapable of DNA binding; without and with induced hyperthyroidism (hyper).

		**Group Size**	**Baseline**	**Pacer Baseline**	**isch5**	**isch25**	**rep10**	**rep20**	**rep30**	**rep40**	**rep50**	**rep60**
CF [mL/min]	control	10	2.4 ± 0.9	2.7 ± 0.9	0.0 ± 0.0 #	0.0 ± 0.0 #	2.9 ± 1.0	2.9 ± 1.3	2.9 ± 1.3	2.8 ± 1.4	2.8 ± 1.4	2.7 ± 1.3
	hypo	7	1.3 ± 0.3 *	1.1 ± 0.3 *	0.0 ± 0.0 #	0.0 ± 0.0 #	2.0 ± 0.8	1.5 ± 0.6	1.4 ± 0.6	1.5 ± 0.5	1.4 ± 0.5	1.4 ± 0.5
	hypo + epi	7	§ 2.2 ± 0.6		0.0 ± 0.0 #	0.0 ± 0.0 #	2.9 ± 1.0	3.0 ± 0.8	3.0 ± 0.8	3.0 ± 0.8	3.0 ± 0.8	2.9 ± 0.8
	hyper	8	3.0 ± 0.9	2.9 ± 0.8	0.0 ± 0.0 #	0.0 ± 0.0 #	2.4 ± 0.6	2.3 ± 0.6	2.3 ± 0.6	2.3 ± 0.7	2.2 ± 0.7	2.2 ± 0.6
	WT	11	2.4 ± 0.9	2.7 ± 0.9	0.0 ± 0.0 #	0.0 ± 0.0 #	2.9 ± 1.0	2.9 ± 1.3	2.9 ± 1.3	2.8 ± 1.4	2.8 ± 1.4	2.7 ± 1.3
	TRα^0^	7	1.6 ± 0.9	1.6 ± 1.0	0.0 ± 0.0 #	0.0 ± 0.0 #	1.5 ± 0.9	1.5 ± 0.9	1.5 ± 0.9	1.6 ± 0.9	1.6 ± 0.8	1.7 ± 0.9
	TRα^0^hyper	5	2.4 ± 0.4	2.6 ± 0.3	0.0 ± 0.0 #	0.0 ± 0.0 #	2.9 ± 0.6	2.8 ± 0.5	2.8 ± 0.4	2.7 ± 0.4	2.5 ± 0.4	2.4 ± 0.4
	TRα^GS^	8	2.5 ± 1.5	2.6 ± 1.2	0.0 ± 0.0 #	0.0 ± 0.0 #	2.9 ± 1.4	2.8 ± 1.4	2.7 ± 1.4	2.7 ± 1.4	2.6 ± 1.4	2.6 ± 1.3
	TRα^GS^hyper	6	1.9 ± 0.5	2.0 ± 0.5	0.0 ± 0.0 #	0.0 ± 0.0 #	2.2 ± 0.6	2.1 ± 0.6	2.1 ± 0.6	2.1 ± 0.7	2.1 ± 0.7	2.0 ± 0.6
		**Group Size**	**Baseline**	**Pacer Baseline**	**isch5**	**isch25**	**rep10**	**rep20**	**rep30**	**rep40**	**rep50**	**rep60**
LVDP [mmHg]	control	10	88 ± 14	91 ± 19	0 ± 0 #	0 ± 0 #	11 ± 19 #	35 ± 27 #	42 ± 28 #	42 ± 27 #	43 ± 26 #	43 ± 24 #
	hypo	7	48 ± 12 *	30 ± 8 *	0 ± 0 #	0 ± 0 #	20 ± 14	23 ± 12	27 ± 15	26 ± 15	29 ± 16	29 ± 17
	hypo + epi	7	§ 97 ± 15		0 ± 0 #	0 ± 0 #	63 ± 24 *	69 ± 22	76 ± 21	77 ± 21	75 ± 19	73 ± 17
	hyper	8	122 ± 22 *	110 ± 20	0 ± 0 #	0 ± 0 #	2 ± 2 #	9 ± 22 #	16 ± 31 #	22 ± 33 #	25 ± 35 #	25 ± 34 #
	WT	11	88 ± 14	91 ± 19	0 ± 0 #	0 ± 0 #	11 ± 19 #	35 ± 27 #	42 ± 28 #	42 ± 27 #	43 ± 26 #	43 ± 24 #
	TRα^0^	7	104 ± 25	55 ± 23 *	0 ± 0 #	0 ± 0 #	40 ± 27 *	47 ± 21 *	49 ± 19	50 ± 17	52 ± 16	51 ± 15
	TRα^0^hyper	5	104 ± 11	96 ± 9	0 ± 0 #	0 ± 0 #	18 ± 20 #	53 ± 23 *#	61 ± 21 *#	63 ± 14 *#	64 ± 10 *#	64 ± 7 *#
	TRα^GS^	8	106 ± 19	90 ± 21	0 ± 0 #	0 ± 0 #	73 ± 25 *	82 ± 19 *	81 ± 19 *	80 ± 15 *	79 ± 14 *	76 ± 14 *
	TRα^GS^hyper	6	84 ± 24	81 ± 20	0 ± 0 #	0 ± 0 #	16 ± 14 #	31 ± 28 #	40 ± 28 #	43 ± 28 #	45 ± 28 #	46 ± 27 #

## Data Availability

All data are available from the corresponding author upon request.

## Supplementary Materials

The following supporting information can be downloaded at: https://www.mdpi.com/article/10.3390/ijms232113340/s1.
