# Supplementary material for: Cardioprotection by Hypothyroidism Is Not Mediated by Favorable Hemodynamics—Role of Canonical Thyroid Hormone Receptor Alpha Signaling
Source: Int J Mol Sci. 2022 Nov 1;23(21):13340. doi: 10.3390/ijms232113340 (PMC9656281; doi:10.3390/ijms232113340)
Supplement: Supplementary file 1 [file ijms-23-13340-s001.zip › ijms-1906449-supplementary.pdf]

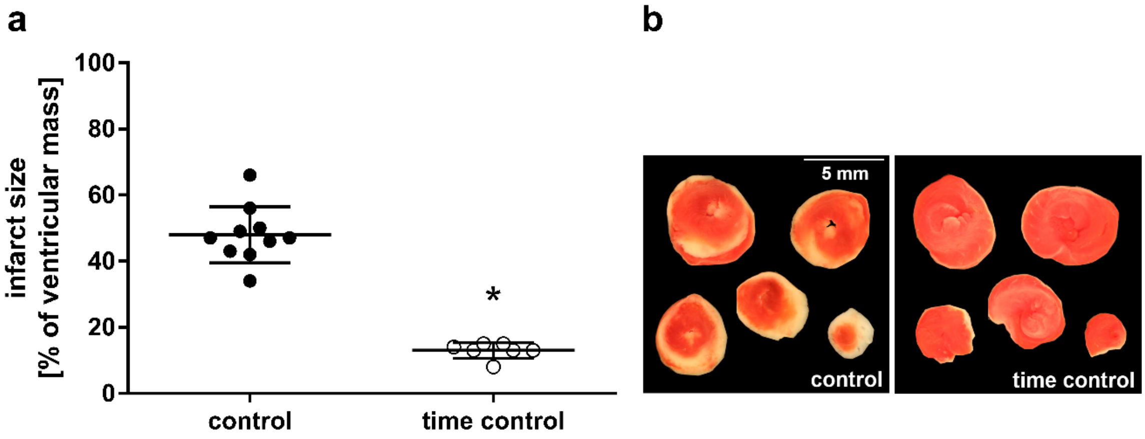

Figure S1. Myocardial infarct size of time controls. Infarct size of isolated pressure constant perfused hearts of control mice received 30/120 global ischemia/reperfusion and time controls (TC). (**a**) Infarct size in [% of ventricular mass] and (**b**) representative heart slices after TTC staining, respectively; n=7-10; Data are means ± standard deviations. * $p < 0.05$ vs. control.

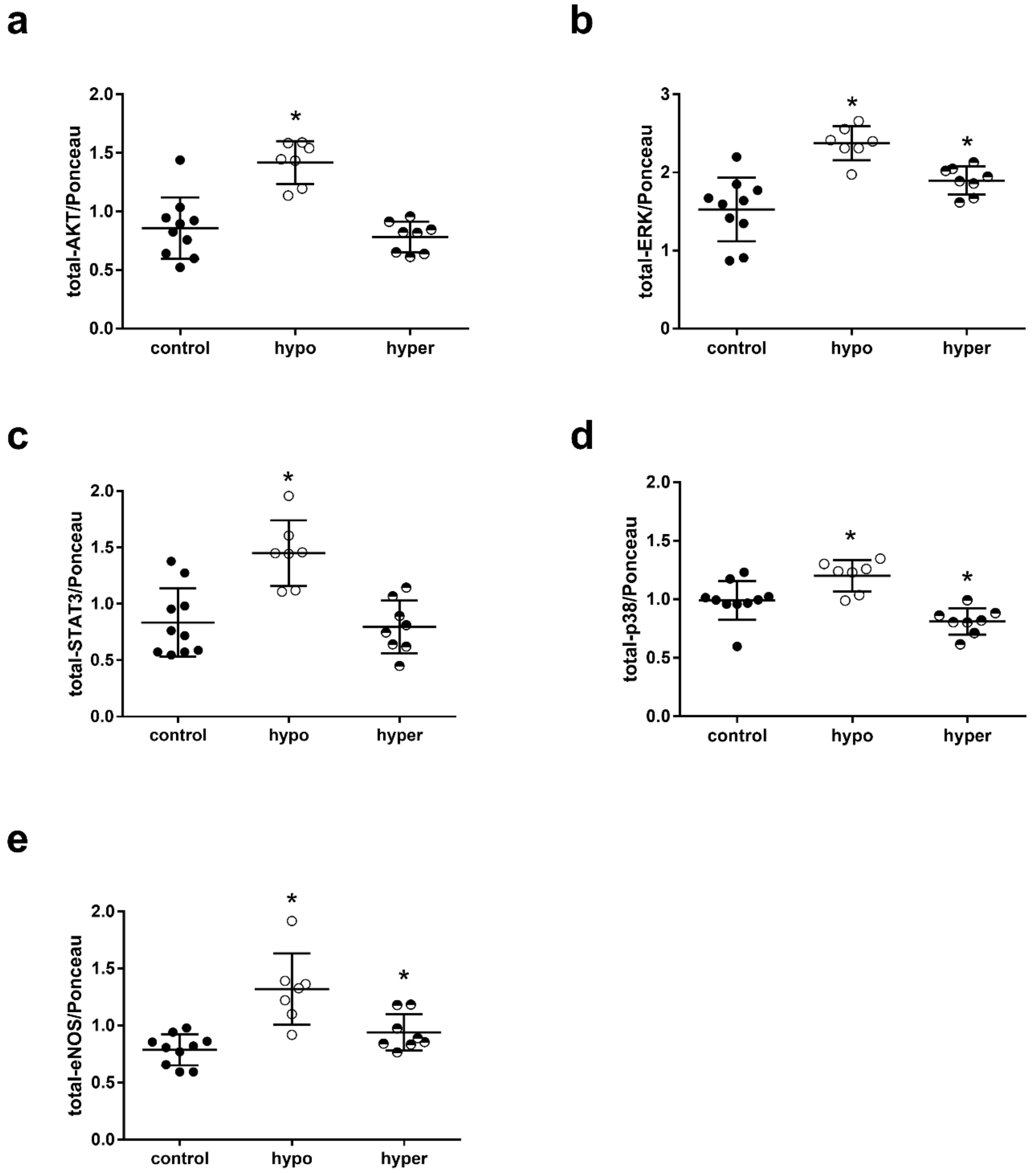

Figure S2. Expression of classical cardioprotective proteins in mice with thyroid dysfunction. Expression of (**a**) protein kinase B (total-AKT/Ponceau), (**b**) extracellular-signal regulated kinases (total-ERK/Ponceau), (**c**) signal transducer and activator of transcription 3 (total-STAT3/Ponceau), (**d**) p38-mitogen-activated protein kinase (total-p38/Ponceau) and (**e**) endothelial nitric oxide synthase (total-eNOS/Ponceau) after 30/120 ischemia/reperfusion. The expression of proteins was normalized to Ponceau S staining; n=7-10; Data are means ± standard deviations; * $p < 0.05$ vs. control.

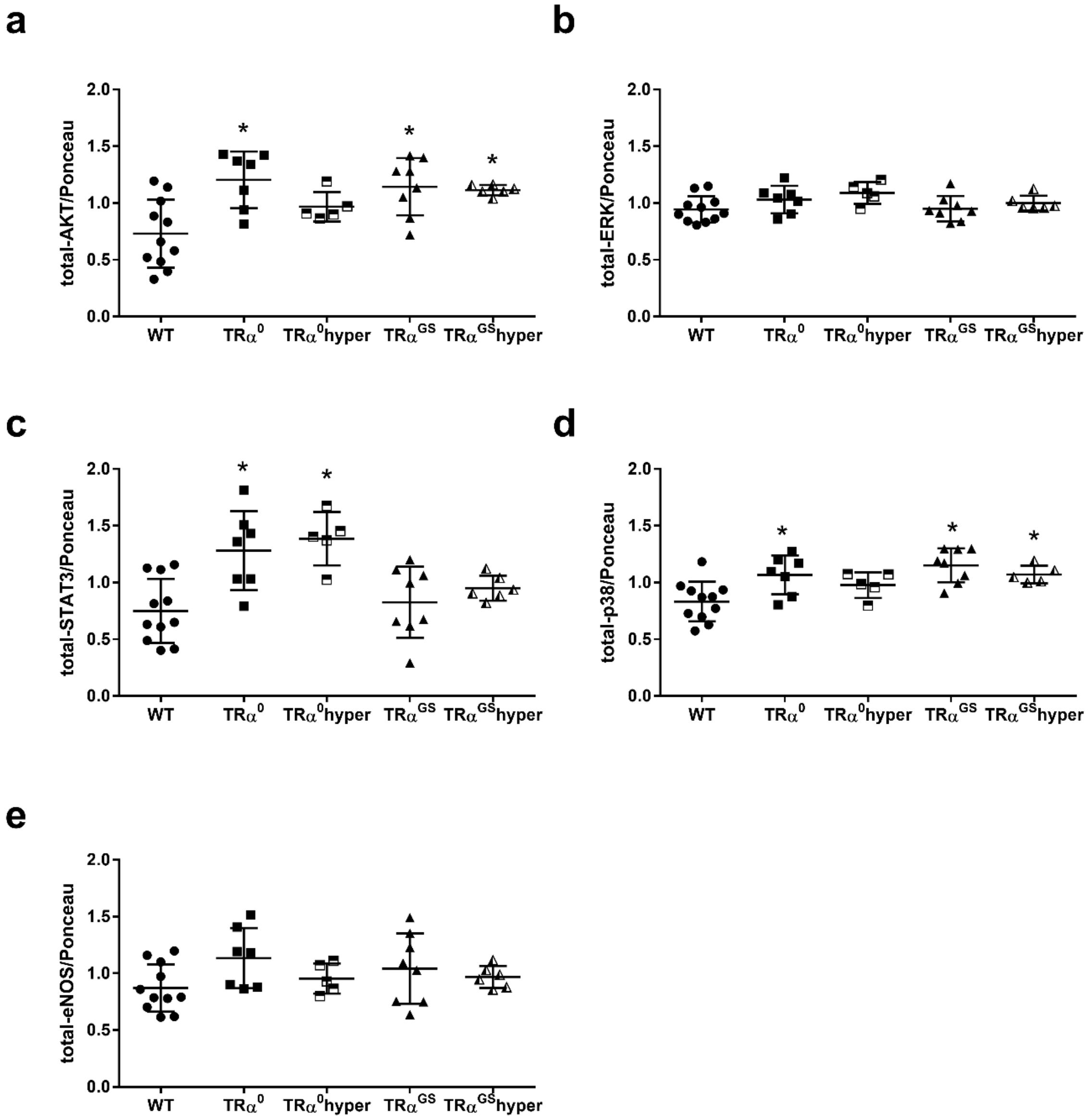

Figure S3. Expression of classical cardioprotective proteins in transgenic mice. Expression of (**a**) protein kinase B (total-AKT/Ponceau), (**b**) extracellular-signal regulated kinases (total-ERK/Ponceau), (**c**) signal transducer and activator of transcription 3 (total-STAT3/Ponceau), (**d**) p38-mitogen-activated protein kinase (total-p38/Ponceau) and (**e**) endothelial nitric oxide synthase (total-eNOS/Ponceau) after 30/120 ischemia/reperfusion. The expression of proteins was normalized to Ponceau S staining; n=5-11; Data are means ± standard deviations; * $p < 0.05$ vs. wildtype (WT).

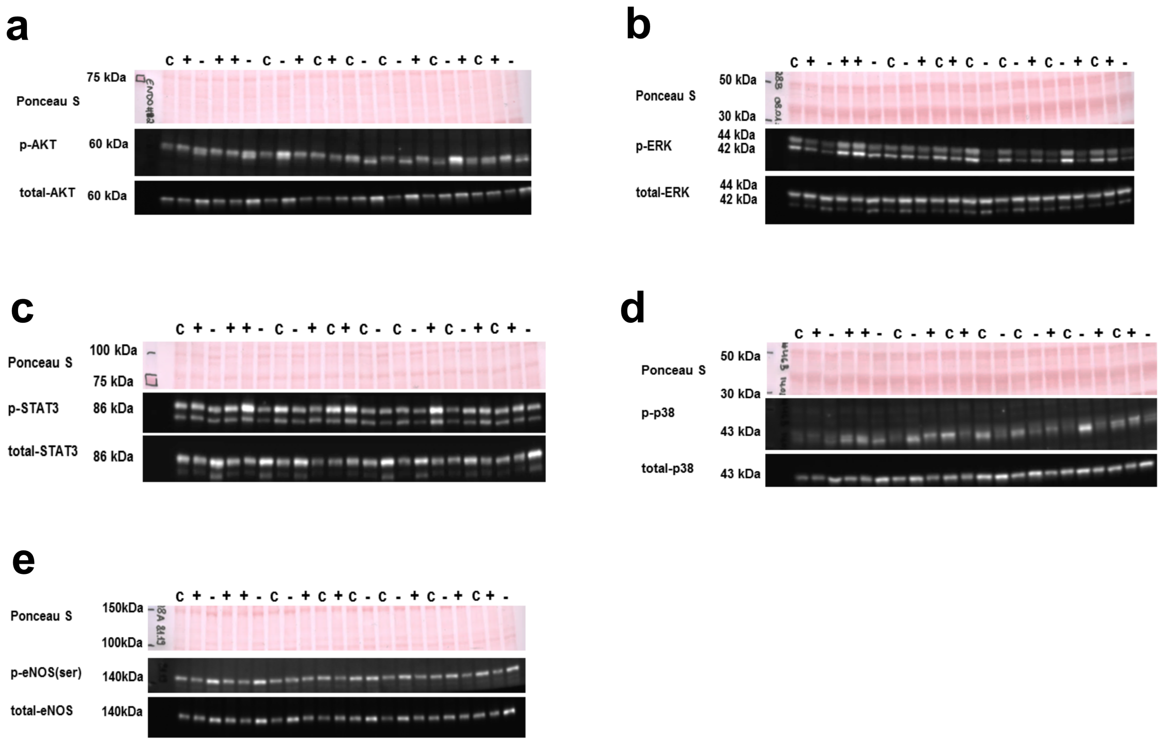

Figure S4. Exemplary Western blots showing expression and phosphorylation of classical cardioprotective proteins in mice with thyroid dysfunction. Top, middle, bottom: Staining with Ponceau S, immunoreactivity signals for phosphorylated and total protein. Expression of (**a**) protein kinase B (AKT), (**b**) extracellular-signal regulated kinases (ERK), (**c**) signal transducer and activator of transcription 3 (STAT3), (**d**) p38-mitogen-activated protein kinase (p38) and (**e**) endothelial nitric oxide synthase (eNOS) after 30 min global ischemia followed by 120 min reperfusion (c indicates control; - hypo and + hyper). The expression of proteins was normalized to Ponceau S staining and expression of phospho-proteins was normalized to total protein expression.

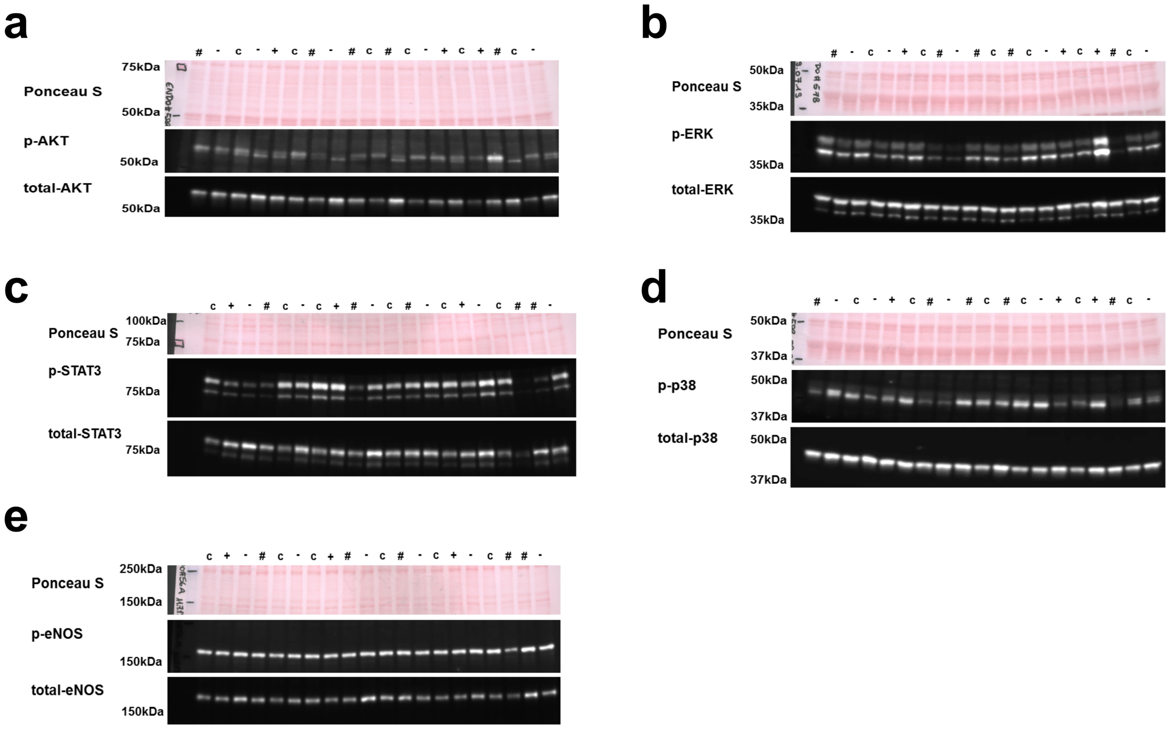

Figure S5. Exemplary Western blots showing expression and phosphorylation of classical cardioprotective proteins in transgenic mice. Top, middle, bottom: Staining with Ponceau S, immunoreactivity signals for phosphorylated and total protein. Expression of (**a**) protein kinase B (AKT), (**b**) extracellular-signal regulated kinases (ERK), (**c**) signal transducer and activator of transcription 3 (STAT3), (**d**) p38-mitogen-activated protein kinase (p38) and (**e**) endothelial nitric oxide synthase (eNOS) after 30 min global ischemia followed by 120 min reperfusion (c indicates wildtype (WT); - $TR\alpha^0$, + hyperthyroid $TR\alpha^0$ ($TR\alpha^0$hyper) and # $TR\alpha^{GS}$). The expression of proteins was normalized to Ponceau S staining and expression of phospho-proteins was normalized to total protein expression.

Table S1. Functional recovery of coronary flow and left ventricular developed pressure in time controls. Functional recovery of coronary flow and left ventricular developed pressure in control mouse hearts that underwent global IR and in time controls that did not underwent global IR. Mean coronary flow (CF) and mean left ventricular developed pressure (LVDP) of control and time control mouse hearts. CF and LVDP were analyzed at different time points: at pacer baseline, at 5 and 25 min of ischemia (isch5, isch25) and at 10, 20, 30, 40, 50, 60 min of reperfusion (rep10-rep60), respectively; n=7-10; Data are means ± standard deviations. * $p < 0.05$ vs. control; # $p < 0.05$ vs. pacer baseline.

Protocol	Time	CF [ml/min]	LVDP [mmHg]
**control**<br>n = 10	pacer baseline	2.7 ± 0.9	91 ± 19
	isch5	0.0 ± 0.0 #	0 ± 0 #
	isch25	0.0 ± 0.0 #	0 ± 0 #
	rep10	2.9 ± 1.0	11 ± 19 #
	rep20	2.9 ± 1.3	35 ± 27 #
	rep30	2.9 ± 1.3	42 ± 28 #
	rep40	2.8 ± 1.4	42 ± 27 #
	rep50	2.8 ± 1.4	43 ± 26 #
	rep60	2.7 ± 1.3	43 ± 24 #
**time control**<br>n = 7	pacer baseline	2.6 ± 0.4	90 ± 15
	isch5	2.5 ± 0.4 #	88 ± 11 *
	isch25	2.4 ± 0.3 #	84 ± 13 *
	rep10	2.4 ± 0.4	82 ± 8 *
	rep20	2.4 ± 0.4	83 ± 11 *
	rep30	2.4 ± 0.4	78 ± 15 *
	rep40	2.3 ± 0.5	75 ± 14 *
	rep50	2.4 ± 0.5	81 ± 18 *
	rep60	2.3 ± 0.5	78 ± 17 *

Table S2. Modified Krebs-Henseleit-buffer for isolated mouse hearts.

**Chemicals**	**mmol/L**
$CaCl_2$	1.6
$C_3H_3NaO_3$	2
$C_6H_{12}O_2$	5.55
EDTA	0.07
KCl	4.7
KH_2PO_4	1.18
$MgSO_4$ x 7 H_2O	1.64
NaCl	118
$NaHCO_3$	24.88

The buffer was filtered through a 0.45 µm filter (Filter Type HWAP, Merck, Darmstadt, Germany), oxygenated (95% O_2 and 5% CO_2) and equilibrated to 37°C before use.
